# Factors associated with anxiety symptoms among medical laboratory professionals in Khobar: Single institution study

**DOI:** 10.3389/fpubh.2022.917619

**Published:** 2022-09-09

**Authors:** Arwa Althumairi, Noot Mishal Ayed AlOtaibi, Arwa Alumran, Saja Alrayes, Amani Owaidah

**Affiliations:** ^1^Department of Health Information Management and Technology, College of Public Health, Imam Abdulrahman bin Faisal University, Dammam, Saudi Arabia; ^2^Department of Clinical Laboratory Sciences, College of Applied Medical Sciences, Imam Abdulrahman bin Faisal University, Dammam, Saudi Arabia

**Keywords:** anxiety level, laboratory, COVID-19, mental status, pandemic, PPE

## Abstract

**Background:**

A clinical professional has a challenging role during the coronavirus disease (COVID-19) pandemic in providing timely and accurate results with limited resources and a rapid increase in the number of samples to be tested. However, during the ongoing pandemic, the anxiety level of Medical Laboratory Professionals (MLT) has not been studied in Saudi Arabia within the hospital environment.

**Aim:**

To determine the associated factors related with anxiety level of MLT at King Fahd Hospital of the University (KFHU) during the COVID-19 pandemic.

**Methods:**

The study design was a cross-sectional quantitative study. Data were collected by administering a paper-based questionnaire that was distributed among MLT at KFHU. The questionnaire consisted of three sections. The first two sections were prepared by the researchers and included participant demographics and questions related to COVID-19. The third section used the Hamilton Anxiety Scale to assess anxiety levels.

**Result:**

The study revealed that 70.4% of MLT showed no signs of anxiety, while 19.2% showed mild/moderate anxiety levels, and approximately 10.4% showed severe to extreme anxiety levels. In addition, a significant association between the anxiety level and difficulty breathing among MLT wearing personal protective equipment was observed. Moreover, a significant association between sex and anxiety level was identified. Females MLT had higher percentages of severe anxiety (12.8% vs. 5.1%) and mild/moderate anxiety (24.4 vs. 7.7%) than males.

**Conclusions:**

Protecting the mental health status of MLT is an essential part of public health measures to fight the COVID-19 pandemic.

## Introduction

The coronavirus disease 2019 (COVID-19) emerged from Wuhan, China, in December 2019 and has spread across the globe rapidly (1). In March 2020, the World Health Organization (WHO) announced COVID-19 as a global pandemic (1). The first confirmed case of COVID-19 in Saudi Arabia was reported on March 3, 2020 (2). Since then, health care providers have been under considerable pressure due to direct and indirect contact with patients with COVID-19 (3). The occurrence of mistakes is expected due to the unpredicted occurrence of the pandemic and its treatment (4). The pressure might be explained by the mental, psychological and physical pressure in terms of getting infected or spreading the virus to their loved ones. Social isolation increases the demand for health care services and work burnout (5–7). Health care providers (HCPs) in pandemic outbreak conditions fight at the frontline either directly (e.g., physicians, nurses) or indirectly (e.g., laboratory technologists) to deliver patient care. Therefore, the HCP workforce plays a critical role in successfully responding to pandemic situations (8, 9).

A cross-sectional study using online questionnaires was performed in China to assess the mental health status of HCPs and administrative staff in hospitals. A total of 2,299 participants were enrolled in the study, 2,042 of which were HCPs, and the remaining 257 were administrative staff. Findings revealed that both groups showed different levels of severity of fear, anxiety, and depression (10). Moreover, comparing administrative staff, HCPs, and frontline HCPs, the results showed that frontline HCPs in close contact with infected patients recorded higher scores for fear, anxiety, and depression (10). However, it was found that the infectious disease was spread through co-worker rather than direct contact with patient (11).

Frontline HCPs were more susceptible to developing mental health disorders (10). This increased susceptibility may be due to long work hours, the need to wear personal protective equipment (PPE), and the routine donning and doffing of full PPE, which results in physical fatigue, mental stress and difficulty breathing (12, 13). Additionally, many HCPs felt unprepared to deal with the new pandemic due to their lack of knowledge. The fear of spreading the virus to families, friends, or colleagues contributed to the overall anxiety (14), prompting HCPs to isolate themselves from their families and change their daily routines. All of these factors affect mental health status through feelings of loneliness, physical and mental fatigue, insomnia and anxiety (14, 15).

Studies supported that wearing masks might have the potential to reduce the risks of COVID-19 and other respiratory diseases; however, proper handling of masks is not known in society (16). Furthermore, previous studies found that using masks as precautionary measures might have mental and physical side effects, such as headache, fever, and difficulty breathing (13, 17). An understanding of the intensity, chronological, social influence, and timing of the pandemic will help in planning for services and help in future research to understand the geographical distribution of such a pandemic (18).

Several factors might affect mental health among health care providers and general populations during COVID-19, including sex differences, age, exposure to social media, news concerning COVID-19, and fake news, which were highly associated with increasing anxiety levels (19). Moreover, fewer years of work experience indicates a greater likelihood of developing mental health challenges as anxiety levels increase (20). One study reported that marital status affected the mental health status, and married individuals had higher anxiety levels than unmarried individuals (21). Conversely, another study found that widows/divorced individuals experienced more anxiety symptoms than married and single individuals (22). Long quarantine periods and contact with patients with COVID-19 or contaminated objects also led to a higher risk of anxiety (23). More studies on mental health during pandemics should be performed to cover different populations at an international level and ensure that appropriate measures are taken to optimize health care worker mental health and thus achieve optimal health care delivery (20).

During the pandemic, medical laboratory technicians (MLT) experienced an increasing demand to collect and analyze specimens in a timely manner with limited resources (24, 25). To the best of our knowledge, studies assessing the mental health status of HCPs during pandemics are limited, and of those studies agreed that MLT are experiencing high stress, anxiety and depression during the pandanis (26–28). The phycological distress could also related with work burn out. There could be several factors influencing psychological status, namely anxiety these could be, age, gender, marital status, educational level and work experience (26, 27). In addition, MLT were not included in most of the previous studies conducted in Saudi Arabia or Middle East despite their critical roles in collecting, handling, and analyzing patient samples, as well as reporting test results. The aim of this study was to determine the anxiety level of MLT at King Fahd Hospital of the University during the COVID-19 pandemic and to determine the factors that influenced their mental health status.

## Research methodology

### Research design

A quantitative cross-sectional study was conducted to determine the factors related with anxiety level among MLT at King Fahd Hospital of the University (KFUH) in Khobar, Saudi Arabia.

### Participants

All MLT from KFUH participated in the study. The sample size was estimated using g power, with 95% confidence interval and level of error is 0.05 was 134 (29). One hundred fifty paper-based questionnaires were distributed to all MLT working in the studied hospital between October 29 and November 15, 2020. All MLT staff (150) at the study hospital were all included in the study and only completed questionnaires (125) were included in the study.

### Instruments

The questionnaire was designed in English and consisted of three sections, and the first two sections were designed and reviewed by the research team. The first section focused on collecting demographic data, which included five sets of close-ended questions on sex, age, marital status, years of experience, and working hours per day. The second section contained the COVID-19-related questions, including three sets of polar questions adapted from a previous study (3). The third section was the Hamilton Anxiety Scale (HAMA), a short scale developed in 1,959 by Dr. M. Hamilton, specified for assessing anxiety level. This tool was firstly design for clinical/diagnostic purposes for patient with anxiety neurosis, then it been commonly used as self-rated anxiety level (30). HAMA was previously assessed for its validity internationally including Arab country the internal validity considered as sufficient were between Cronbach alpha = 0.90–0.92 (31, 32). The third section contained 14 questions. The scoring was graded from 0 to 5 points as follows: 0 (not present), 1 (mild), 2 (moderate), 3 (severe), and 4 (very severe). Then, the total HAMA score was categorized as severe anxiety (score ≥ 14 points), mild and moderate anxiety (score of 7–13 points), and no anxiety (score of 0–6 points) following the guideline of study with similar geographical area (10, 33). The questionnaire survey was distributed in English as participant where fluent in English as shown in Appendix 1.

### Ethics

Ethical approval was obtained from the Institutional Review Board of Imam Abdulrahman Bin Faisal University, IRB Number: IRB-PGS-2020-03-350 (Appendix II). Additionally, this paper follows the principles of the Declaration of Helsinki and STROBE guidelines.

### Analysis

All statistical analyses were performed using Statistical Package for Social Sciences (SPSS), Version 27.0. Armonk, NY. A chi-square test (X^2^) was performed in this study to assess the significance of associations between exposure factors (sex, age, marital status, years of experience, and working hours per day) and the outcome (anxiety scale). The results were considered statistically significant at a *p* < 0.05.

## Results

### Participant characteristics

Eighty-three percent of MLT responded to the survey and were eligible, for a total of 125 participants; their characteristics are described in Table 1. Approximately 68.8% of the participants were females, while the males represented 31.2% of the participants. The majority of the participants were married (72.8%). Approximately 78.40% of the participants were aged <40 years. The majority of the participants had 6–11 and 11–15 years of experience at 33.60 and 30.40%, respectively. Moreover, 72.8% of the total participants worked an 8 h shift (Table 1).

**Table 1:** Participant characteristics.

**Participant characteristics**	**Number of participants N = 125**	**%**
**Sex**		
Male	39	31%
Female	86	69%
**Marital status**		
Single	33	26%
Married	91	73%
Others	1	1%
**Age, years**		
≤ 25	5	4%
26–30	22	18%
31–35	36	29%
36–40	35	28%
≥41–45	17	14%
46	10	8%
**Years of experience**		
≤ 5 years	23	18%
6–10 years	42	34%
11–15 years	38	30%
≥16 years	22	18%
**Working hours**		
≤ 8 hours	34	27%
> 8 hours	91	73%
**Diagnosed with COVID-19**		
Yes	13	10%
No	112	90%
**Managed a COVID-19 sample**		
Yes	62	50%
No	63	50%
**Wearing PPE caused you breathing difficulty (%)**		
Yes	40	32%
No	85	68%

### Exposure to COVID-19

As shown in Table 2 most of the MLT were not diagnosed with COVID-19 (89.60%). Additionally, ~50.40% of the MLT managed or processed a COVID-19 sample. Additionally, ~32% of the MLT reported difficulty breathing while wearing PPE.

**Table 2:** Comparison of anxiety levels among participants with different demographic characteristics.

**Participant characteristics**	**Number of participants**		**Anxiety status**		**Chi-square**	
		**No anxiety**	**Mild and moderate anxiety**	**Severe anxiety**	**X^2^**	***P* value**
	***N* = 125**	***N* = 88 (70%)**	***N* = 24 (19%)**	***N* = 13 (10)**		
Sex (%)						
Male	39	34 (87.2%)	3 (7.7%)	2 (5.1%)	7.692	0.021^*^
Female	86	54 (62.8)	21 (24.4%)	11 (12.8%)		
Marital status (%)						
Single	33	19 (57.6%)	10 (30.3%)	4 (12.1%)	4.359	0.36o
Married	91	68 (74.7%)	14 (15.4%)	9 (9.9%)		
Others	1	1 (100%)	0 (0.0%)	0 (0.0%)		
Age, years (%)						
**≤** 25	5	4 (80%)	0 (0.0%)	1 (20.0%)	3.508	0.967
26–30	22	15 (68.2%)	6 (27.3%)	1 (4.5%)		
31–35	36	26 (72.2%)	6 (16.7%)	4 (11.1%)		
36–40	35	25 (71.4%)	6 (17.1%)	4 (11.4%)		
41–45	17	11 (64.7%)	4 (23.5%)	2 (11.8%)		
**≥** 46	10	7 (70.0%)	2 (20.0%)	1 (10.0%)		
Years of experience (%)						
**≤** 5 years	23	14 (60.9%)	6 (26.1%)	3 (13.0%)	4.363	0.628
6–10 years	42	30 (71.4%)	9 (21.4)	3 (7.1%)		
11–15 years	38	26 (68.4%)	6 (15.8%)	6 (15.8%)		
**≥**16 years	22	18 (81.8%)	3 (13.6%)	1 (4.5%)		
Working hours (%)						
**≤**8 h	34	24 (70.6%)	7 (20.6%)	3(8.8%)	0.159	0.924
**>**8 h	91	64 (70.3%)	17 (18.7%)	10 (11.0%)		
Diagnosed with COVID-19 (%)						
Yes	13	6 (46.2%)	4 (30.8%)	3 (23.1%)	4.465	0.107
No	112	82 (73.2%)	20 (17.9%)	10 (8.9%)		
Managed COVID-19 samples (%)						
Yes	62	43 (69.4%)	14 (22.6%)	5 (8.1%)	1.397	0.497
No	63	45 (71.4%)	10 (15.9%)	8 (12.7%)		
Wearing PPE caused difficulty breathing (%)						
Yes	40	20 (50.0%)	11 (27.5%)	9 (22.5%)	13.869	0.001^*^
No	85	68(80.0%)	13(15.3%)	4(4.7%)		

* P < 0.05.

^**^P < 0.001.

### The anxiety level of participants

The anxiety level of MLT during COVID-19 was explored, as shown in; 70.4% were classified as not having anxiety, 19.2% were classified as having mild/moderate anxiety levels, and approximately 10.4% were classified as having severe to extreme anxiety levels.

### Comparison of anxiety levels based on demographic characteristics

As shown in Table 2, a statistically significant association between the anxiety level and sex was observed [X^2^ (DF) = 7.692 (2), *p* < 0.05]. Females had a higher percentage of severe anxiety (12.8%) than males (5.1%), and 24.4% of females had mild/moderate anxiety was compared with only 7.7% of male. MLT who considered wearing PPE to cause difficulty breathing had higher anxiety levels (22.80%) and ~(27.5%) were classified as having mild and moderate anxiety levels.

A statistically significant association was observed between the anxiety level and difficulty of breathing while wearing PPE [X^2^ (DF) = 13.869 (2), *p* < 0.05], (Table 2). No associations were observed between marital status, age, years of experience, working hours, COVID-19 diagnosis, and managing COVID-19 samples (all *P* > 0.05).

## Discussion

HCPs are often imperiled due to exposure to different types of professional hazards within their workplaces, especially infectious diseases (34). Studying the anxieties of HCPs during the current ongoing COVID-19 pandemic may provide important lessons for managing future pandemics (35, 36).

To the best of our knowledge, this study is the first to focus on the anxiety levels of MLT during the COVID-19 pandemic and related factors. The majority of participants experienced no or mild anxiety. The current study focused on a group that was not studied before and required further intervention to maintain their psychological health.

In the present study, a statistically significant association was observed between anxiety levels and the number of MLT who experienced difficulty breathing while wearing PPE. This finding might be explained by changes in the laboratory guidelines to wear masks to abide by COVID-19 protective and social distancing protocols, while masks are often not necessary in routine practice. Many studies have found that due to the extensive increase in demand for HCPs, they experience long work hours and routinely don and doff full PPE, including face masks, gowns, and gloves, which increase physical fatigue, mental stress, social isolation and difficulty breathing (12, 37, 38).

Moreover, this study also revealed a correlation between the anxiety status and sex, and a statistically significant correlation between sex and the anxiety level was identified. A higher percentage of female MLT had mild to moderate anxiety levels than males. This finding was similar to previous studies conducted during the severe acute respiratory syndrome (SARS) epidemic. Females had higher stress levels and higher levels of depression and anxiety, and they also experienced more severe posttraumatic stress symptoms (39). Likewise, systematic research on the prevalence of symptoms and factors associated with mental health in the general population during the COVID-19 pandemic revealed an association in females exposed to the news concerning COVID-19. The sex differences and mental health have been explained in an earlier study, in which females had a lower ability to cope with trauma than males due to their biological and psychological nature (40). Sex is an important variable that should be considered in biological and social science studies, and more in-depth research must be conducted to understand the causes of these differences (41).

Fake news was strongly associated with increased anxiety levels. Exposure to news *via* different media sources, such as TV, Twitter and other channels, was significantly related to increased stress and anxiety among populations that reflected the health care provider's mental wellbeing (21, 23). Additionally, anxiety levels have increased not only by exposure to fake news but also by misunderstandings of the reported incidence and fatality rate of COVID-19 by the public (42). Saudi Arabia limited the channels that reveal the number of COVID-19 incidences only through authorized parties such as the Ministry of Health (MOH) websites, official social media accounts and MOH press conferences to ensure accuracy and transparency and to control what information is presented in the media.

A study conducted by Lai et al. (20) revealed that fewer years of work experience rendered individuals more likely to develop mental health illnesses due to increased anxiety levels (20). Exposing to a change in workforce or uncertainty in work, longer work hours usually occurred to joiner staff in which might increase anxiety (20, 43). Additionally, marital status affects mental health status, and married individuals have higher anxiety levels than unmarried individuals (21), and that could be justified as the fear of spread the disease to their beloved once (44). Due to the limited number of participants in our study, the association between age, marital status, years of experience, working hours and anxiety level have no significant finding.

Despite the limited number of participants in the study, only one group of health care providers was analyzed. This information provides insights into the anxiety status of an important group of health care practitioners that were not included in previous studies. This study should be generalized to include all groups of HCPs from the different regions of Saudi Arabia to accurately assess the mental health status of health care practitioners. A study of the factors that might affect the mental health status of laboratory technicians, such as the type of hospital, namely, public or private hospitals, or laboratory unit section, would be very helpful for implementing changes in a health care setting in Saudi Arabia. Nevertheless, Saudi Arabia made a remarkable effort to contain the pandemic by providing the hospital with well-equipped materials and, more importantly, a quick risk management plan to cope with the pandemic, which had a major effect on reducing the stress among HCP and the general population (45, 46). Although the use of masks helped reduce the spread of the disease, some guidelines were recommended to follow during and beyond the COVID-19 pandemic (47). Another limitation of the study was that patient were not asked about previous history of anxiety, this could be correlated with anxiety level in the baseline level before COVID-19. However, other studied showed that there is no relation of previous exposing to infectious diseases and level of anxiety (48).

## Conclusions

Protecting the mental health status of MLT is an essential part of public health measures to fight the COVID-19 pandemic due to their critical role in handling COVID-19 samples. Furthermore, anxiety level and other mental health status of health care providers during COVID-19 deserves attention, perhaps through online mental therapy clinics or through developing mental health consultation programs to be provided by hospitals in Saudi Arabia.

## Data availability statement

The raw data supporting the conclusions of this article will be made available by the authors, without undue reservation.

## Ethics statement

Ethical approval was obtained from the Institutional Review Board of Imam Abdulrahman Bin Faisal University, IRB Number: IRB-PGS-2020-03-350 (Appendix II). The patients/participants provided their written informed consent to participate in this study.

## Author contributions

AAlt, NA, AAlu, SA, and AO contributed to the design and implementation of the research, to the analysis of the results, and to the writing of the manuscript.

## Conflict of interest

The authors declare that the research was conducted in the absence of any commercial or financial relationships that could be construed as a potential conflict of interest.

## Publisher's note

All claims expressed in this article are solely those of the authors and do not necessarily represent those of their affiliated organizations, or those of the publisher, the editors and the reviewers. Any product that may be evaluated in this article, or claim that may be made by its manufacturer, is not guaranteed or endorsed by the publisher.
