# Supplementary material for: Factors associated with anxiety symptoms among medical laboratory professionals in Khobar: Single institution study
Source: Front Public Health. 2022 Sep 9;10:917619. doi: 10.3389/fpubh.2022.917619 (PMC9500507; doi:10.3389/fpubh.2022.917619)

**Appendix:**

**The Questionnaire (Appendix 1)**

We want to tell you about a research study we are doing. A research study is a way to learn more about something. We would like to learn more about the mental health status of medical laboratory technicians (MLT) during the COVID-19 pandemic. You are being asked to join the study because you are MLT and living in Saudi Arabia.

In response to the global COVID-19 pandemic, we developed this questionnaire to assess mental status in terms of the anxiety level among MLT during COVID-19.

If you complete the questionnaire below, you agree to participate in this research study and agree to share your information for research purposes.

Do you agree to participate:

**Yes No**

| Section A: Demographic section | |
| --- | --- |
| **1.** What is your sex? | □Male □ Female |
| **2.** What is your marital status? | □ Single □ Married □ Others |
| **3.** How old are you? | □≤25 □ 26-30 □ 31-35  □ 36-40 □ 41-45 □≥46 |
| **4.** How many years of experience do you have in your current profession? | □≤ 5 □ 6-10 □ 11-15 □≥16 |
| **5.** How many hours do you work per day? | □ ≤8 hours □>8 hours |
| Section B: COVID-19-related questions | |
| **1.** Have you been diagnosed with COVID-19? | □ Yes □ No |
| **2.** Do you manage with samples from patients diagnosed with COVID-19? | □ Yes □ No |
| **3.** During the COVID-19 pandemic, has personal protective equipment (PPE) caused you breathing difficulties? | □ Yes □ No |

| **Section C:** Below is a list of phrases that describe certain feelings that you might experience during the COVID-19 pandemic. Rate yourself by selecting the answer that best describes the extent to which you had these conditions. Select one of the five responses.  **0 = Not present, 1 = Mild, 2 = Moderate, 3 = Severe, 4 = Very severe** | |
| --- | --- |
| **1. Anxious mood (**Worries, anticipation of the worst, fearful anticipation, irritability). | □ Not present □ Mild □ Moderate  □ Severe □ Very severe |
| **2. Tension (**Feelings of tension, fatigability, startle response, moved to tears easily, trembling, feelings of restlessness, inability to relax). | □ Not present □ Mild □ Moderate  □ Severe □ Very severe |
| **3. Fears** (Of the dark, of strangers, of being left alone, of animals, of traffic, of crowds). | □ Not present □ Mild □ Moderate  □ Severe □ Very severe |
| **4. Insomnia** (Difficulty falling asleep, disrupted sleep, unsatisfying sleep and fatigue on waking, dreams, nightmares, night terrors). | □ Not present □ Mild □ Moderate  □ Severe □ Very severe |
| **5. Intellectual** (Difficulty concentrating, poor memory). | □ Not present □ Mild □ Moderate  □ Severe □ Very severe |
| **6. Depressed mood (**Loss of interest, lack of pleasure in hobbies, depression, early waking, diurnal swing). | □ Not present □ Mild □ Moderate  □ Severe □ Very severe |
| **7. Somatic (muscular) (**Pains and aches, twitching, stiffness, myoclonic jerks, grinding of teeth, unsteady voice, increased muscular tone). | □ Not present □ Mild □ Moderate  □ Severe □ Very severe |
| **8. Somatic (sensory)** (Tinnitus, blurring of vision, hot and cold flushes, feelings of weakness, pricking sensation). | □ Not present □ Mild □ Moderate  □ Severe □ Very severe |
| **9. Cardiovascular symptoms (**Tachycardia, palpitations, chest pain, throbbing of vessels, fainting feelings, missing beat). | □ Not present □ Mild □ Moderate  □ Severe □ Very severe |
| **10. Respiratory symptoms (**Pressure or constriction in chest, choking feelings, sighing, dyspnea). | □ Not present □ Mild □ Moderate  □ Severe □ Very severe |
| **11. Gastrointestinal symptoms** (Difficulty swallowing, abdominal pain, burning sensations, abdominal fullness, nausea, vomiting, borborygmi, looseness of bowels, loss of weight, constipation). | □ Not present □ Mild □ Moderate  □ Severe □ Very severe |
| **12. Genitourinary symptoms** (Frequency of micturition, urgency of micturition, amenorrhea, menorrhagia, development of frigidity, premature ejaculation, loss of libido, impotence). | □ Not present □ Mild □ Moderate  □ Severe □ Very severe |
| **13. Autonomic symptoms (**Dry mouth, flushing, pallor, tendency to sweat, giddiness, tension headache, raising of hair). | □ Not present □ Mild □ Moderate  □ Severe □ Very severe |
| **14. Behavior (**Fidgeting, restlessness or pacing, tremor of hands, furrowed brow, strained face, sighing or rapid respiration, facial pallor, swallowing, etc.). | □ Not present □ Mild □ Moderate  □ Severe □ Very severe |

Appendix II

Copy of the ethical approval certificate


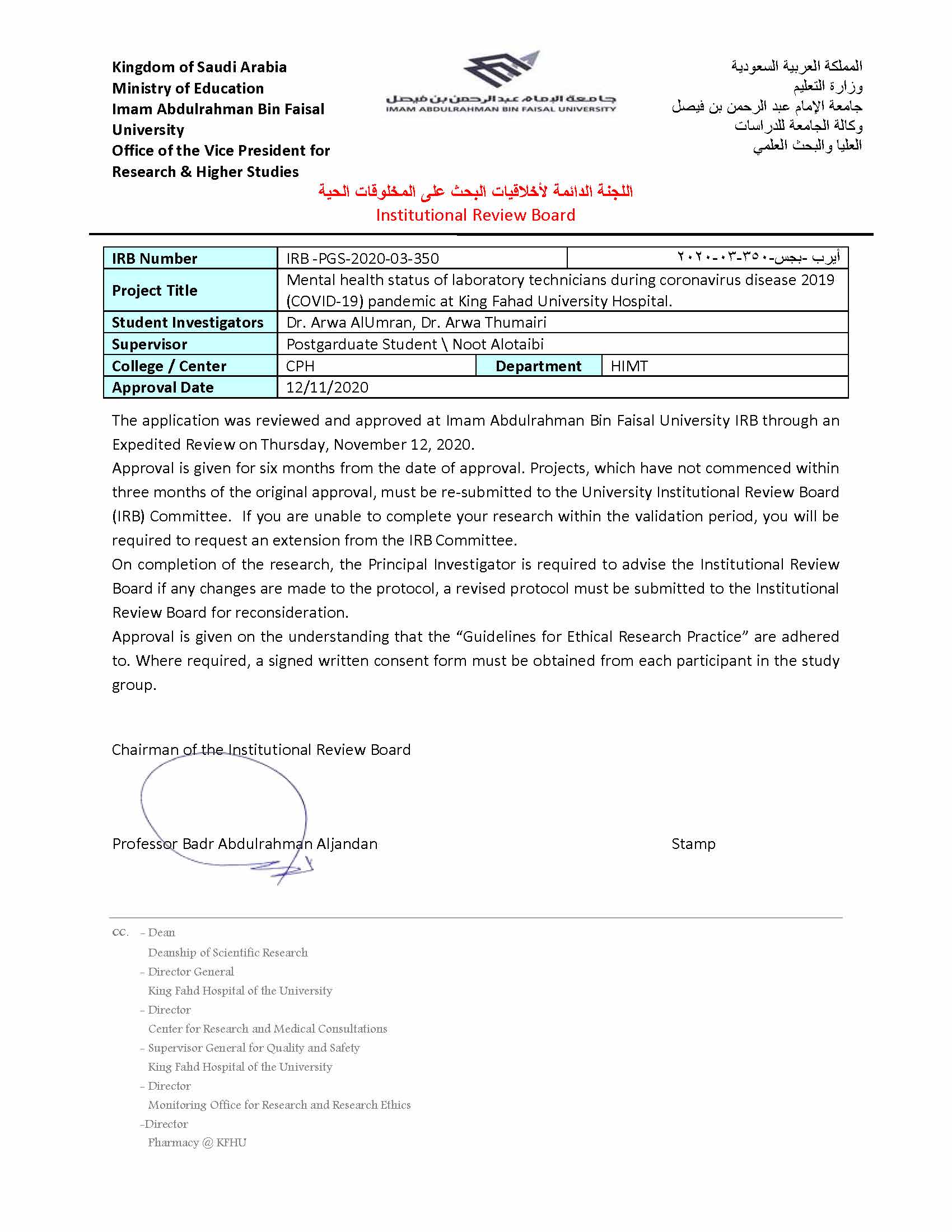

Supplement: Supplementary file 1 [file Data_Sheet_1.docx]
